# Analysis of the epidemiological profile of congenital hip deformities, 2011–2021

**DOI:** 10.1590/1984-0462/2024/42/2022167

**Published:** 2023-07-10

**Authors:** Bianca Gabriella de Oliveira, Fabrício Negreiros Holtz, Matheus Pedroso Cavalcanti de Souza, Nattan Santos Rozendo de Oliveira

**Affiliations:** a Universidade Salvador, Salvador, BA, Brazil.; b Associação de Caridade Hospital Iguaçu, Rio de Janeiro, RJ, Brazil.; c Centro Universitário Facisa, Campina Grande, PB, Brazil.; d Hospital Dom Luiz Gonzaga Fernandes, Campina Grande, PB, Brazil.

**Keywords:** Hip, Congenital hip dysplasia, Congenital hip dislocation, Hospitalization, Quadril, Displasia congênita de quadril, Luxação congênita de quadril, Internação hospitalar

## Abstract

**Objective:**

This study aimed to identify the relevance of hospitalizations for congenital hip deformities in Bahia.

**Methods:**

This is a retrospective epidemiological study in public databases. Descriptors in health sciences: “congenital hip dysplasia”, “congenital hip dislocation”, and “congenital dislocation hip”. This is qualitative-quantitative research with the analysis of secondary data and cross-sectional typologies in the databases of the Ministry of Health – Health Information (TABNET), made available by the Department of Informatics of the Unified Health System (DATASUS).

**Results:**

Bahia was the third Brazilian state with the highest number of hospitalizations, registering 1481 cases. The municipalities in Bahia with the highest prevalence were Itanhém, Salvador, and Barreiras, with 912, 445, and 20 cases, respectively.

**Conclusions:**

The elevated number of congenital hip deformities reflects a public health problem, requiring investments in public policies.

## RESUMO

**Objetivo:**

Identificar a relevância das internações por deformidades congênitas do quadril na Bahia.

**Métodos:**

Este é um estudo epidemiológico retrospectivo, feito em bases de dados públicas. Descritores em ciências da saúde: “displasia congênita de quadril”, “Hip Dislocation Congenital” AND “Luxação congênita de quadril”. Trata-se de uma pesquisa qualiquantitativa, com análise de dados secundários e tipologia transversal nas bases de dados do Ministério da Saúde — Informações de Saúde (TABNET), disponibilizados pelo Departamento de Informática do Sistema Único de Saúde (DATASUS).

**Resultados:**

A Bahia foi o terceiro estado brasileiro com maior número de internações, registrando 1.481 casos. Os municípios baianos com maiores prevalências foram Itanhém, Salvador e Barreiras, com 912, 445 e 20 casos, respectivamente.

**Conclusão:**

O elevado número de deformidades congênitas do quadril reflete um problema de saúde pública, necessitando de investimentos em políticas públicas.

## INTRODUCTION

The concept of “congenital” is linked to everything inherent to birth. Congenital malformations are structural or functional anomalies in which their etiological agent is a factor that originated before birth, whether genetic, environmental, or unknown.^1^

The acetabulofemoral joint, called the hip, consists of four bones (ilium, ischium, pubis, and femur) of the spheroidal diarthrosis type. The femoral head projects directly into the acetabulum for the proper execution of joint movements, namely, flexion, extension, abduction, adduction, and internal and external rotation. Furthermore, to have physiological growth and development, an interdependent relationship between these structures is necessary, which are covered by hyaline cartilage that guarantees anatomic protection and adequate sliding.^2^

When this relationship is compromised, abnormal hip development occurs, which is a very limiting condition for the individual, since the main functions include support of body weight, in addition to transmitting the load from the skeleton to the lower limbs.^2^

Early diagnosis of these congenital lesions allows for a better prognosis. Immediate treatment reduces the impacts generated by the deformities, in addition to generating less pain. Children’s hip pain, which is not always frequent and present, is one of the most common reasons for seeking orthopedic consultation, and the diagnosis is made through medical anamnesis, physical examination, and complementary exams. The painless condition sometimes hinders the diagnosis as it reflects the lack of demand for medical care and/or an adequate workup, leading to a late diagnosis of developmental dysplasia of the hip (DDH). The treatment instituted can be drug, physical therapy, and/or surgical or even conservative treatment through orthosis.^2^

Among the congenital deformities of the hip, it is possible to measure the DDH, an anatomical abnormality with implications for deviation from normal development, during the embryonic, fetal, and child growth period, which may or may not last until puberty. As a result, the interruption of the development of the acetabular cavity causes the non-integrity of the concentric position of the femoral head, which generates a displacement that projects out of the acetabulum, which may be fully displaced or subluxated.^3^

The clinic of congenital hip dysplasia has suggestive signs: asymmetry of the thigh folds and popliteal folds, apparent shortening of the femur, asymmetry of the inguinal folds, and laxity of hip and knee extension.^3^ The diagnosis is made in newborns and infants through the Ortolani and Barlow maneuvers. If the physical examination is inconclusive, hip ultrasound can be used, which allows for accurate visualization of the acetabulofemoral relationship.^4^

In this way, the epidemiological knowledge of congenital hip deformities in Bahia favors the shift of public investments to services aimed at assisting this population, which generates actions for early diagnosis and the immediate initiation of adequate treatment with the reduction of sequelae and their imposed limitations.

## METHOD

This is a retrospective, qualitative-quantitative epidemiological study, whose data were obtained through consultations in the databases of the Ministry of Health — Health Information (TABNET), made available by the Department of Informatics of the Unified Health System, Saúde (DATASUS), accessed throughout the research period. As it is a public domain database, it was not necessary to submit the project to the Research Ethics Committee.

The study population consists of patients hospitalized for congenital hip deformity; this manifestation is being evaluated in both sexes, of all ethnicities, and age groups, in public and private services, from 2011 to 2021, in the state of Bahia. The years with the highest incidence of hospitalizations for congenital hip deformities were measured, and the data were collected from the analysis of the category entitled ICD-10 morbidity list.

The research gathers health data and involves the category “epidemiology and morbidity”, with the selected group “Hospital Morbidity of the SUS (SIH/SUS)”, as well as the option “General, by place of hospitalization – from 2011”, specifying the State of Bahia and its municipalities. In addition to the category “hospitalizations”, the categories “value of hospital services”, “average hospitalization value”, “days of stay”, “deaths”, and “age group” were selected.

The terms were derived from the Health Sciences Descriptors platform. The result includes articles in Portuguese and English. Documentary research was carried out with a literature review based on selected articles in the SciELO, PubMed, and LILACS databases published between 2000 and 2021, using the terms “congenital hip dysplasia”, “congenital hip dislocation”, and “congenital dislocation hip”. The selection criteria for articles included those that address relevant aspects of congenital hip deformities and their epidemiological profile, as well as their definition, risk factors, etiology, symptomatology, pathophysiology, diagnosis, treatment, and complications.

## RESULTS

Between 2011 and 2021, Bahia was the third Brazilian state with the highest number of hospitalizations for congenital hip deformity (Table 1), where 1,481 cases were recorded, which corresponds to 10.2% of notifications in Brazil during the period. The municipality of Itanhém had 61.5% (prevalence=47.4/1,000 inhabitants) of cases registered in Bahia, followed by the city of Salvador with 30.0% (prevalence=0.15/1,000 inhabitants) and Barreiras with 1.3 % (Table 2).^5,6^

**Table 1.** Hospitalizations for congenital hip deformity.^5^

State	Hospital admissions
São Paulo	3644
Paraná	1537
Bahia	1481
Rio Grande do Sul	1292
Minas Gerais	1169
Santa Catarina	1165
Rio de Janeiro	1009

**Table 2.** Estimated population, number of cases, and prevalence of congenital hip deformities in Bahia during the years 2011–2021.^5,6^

	Estimated population	Number of cases	Prevalence
Bahia	14,985,284	1481	9.8/100,000 inhabitants
Itanhém	19,231	912	47.4/1000 inhabitants
Salvador	2,900,319	445	0.15/1000 inhabitants
Barreiras	158,432	20	0.12/1000 inhabitants
Ibicuí	16,682	20	1.19/1000 inhabitants
Feira de Santana	624,104	18	0.028/1000 inhabitants
Vitória da Conquista	343,643	09	0.026/1000 inhabitants
Santo Antônio de Jesus	103,204	09	0.087/1000 inhabitants
Juazeiro	219,544	08	0.036/1000 inhabitants
Guanambi	85,353	07	0.082/1000 inhabitants
Teixeira de Freitas	164,290	06	0.036/1000 inhabitants
Itabuna	214,123	04	0.018/1000 inhabitants
Eunápolis	115,360	03	0.026/1000 inhabitants
Irecê	74,050	03	0.04/1000 inhabitants
Alagoinhas	153,023	02	0.013/1000 inhabitants

Comparing 10 years of study, there was a great disparity between the genders. Women accounted for 84.8% of cases and men for 15.1%, establishing a percentage difference of 69.6%. Of the patients, 70.6% were brown.^6^

The average number of hospital stay per person is 3.2 days. A total of 4,729 days occurred during the study period. The average value per hospitalization was Brazilian real (BRL) 920.40, reaching BRL 1,006.00 in 2016. Thus, hospital expenses in the state were approximately BRL 1,000,500.00 in the last decade. Although the city of Salvador had a lower prevalence when compared to the city of Itanhém, the capital recorded expenditures of BRL 535,378.00, 27.3% higher than Itanhém (Table 3).^6^

**Table 3.** Value of hospital services in cases of congenital hip deformities in Bahia during the years 2011–2021.^6^

	Value of hospital services (Brazilian reais)
Bahia	1,003,456.39
Salvador	535,378.19
Itanhém	389,738.00
Barreiras	7514.00
Ibicuí	7948.00
Feira de Santana	31,518.00
Vitória da Conquista	6665.54
Santo Antônio de Jesus	7246.05
Juazeiro	1413.18
Guanambi	4300.00
Teixeira de Freitas	6356.06
Itabuna	409.40
Eunápolis	308.74
Irecê	834.52
Alagoinhas	192.65

The age groups recorded in the admissions were discrepant. All cases reported in the city of Itanhém were from 10 years of age, with 17.9% of cases (10–19 years old), 47.5% (20–29 years old), and 34.4% (30–49 years old). In the city of Salvador, the records were earlier: 48.5% (0–4 years old), 23% (5–9 years old), 20.8% (10–19 years old), and 7.1% (20–49 years old). In Barreiras, of the 20 registered cases, 85% were younger than 1 year of age and only 15% aged between 20 and 49 years (Table 4).

**Table 4.** Hospitalizations by age group due to congenital hip deformities in Bahia during the years 2011–2021.^6^

County	Less than 1 year old	1 to 4 years old	5 to 9 years old	10 to 14 years old	15 to 19 years old	Above 20 years old
Itanhém	–	–	–	6	158	748
Salvador	49	167	104	67	26	32
Barreiras	17	–	–	–	–	3
Ibicuí	–	–	–	–	2	18
Feira de Santana	7	4	2	3	–	2
Vitória da Conquista	2	–	1	2	–	3
Santo Antônio de Jesus	1	–	1	–	–	7
Juazeiro	–	–	1	–	–	5
Guanambi	1	3	–	1	–	2
Teixeira de Freitas	–	–	–	3	–	3
Itabuna	1	–	–	1	–	2

Regarding the hospitals that most provided care to these patients, there is the Maria Moreira Lisboa Hospital, in the city of Itanhém, with 912 records of assistance to patients with congenital hip deformity. While in the city of Salvador, Martagão Gesteira Hospital and SARAH were responsible for 21.4% of the care provided (Table 5).^6^

**Table 5.** Main establishments helping congenital hip deformities in Bahia during the years 2011–2021.^6^

Establishments	Hospitalizations
Maria Moreira Lisboa Hospital	912
Martagão Gesteira Hospital	215
SARAH Salvador	102
Hospital Santo Antônio	45
Santa Isabel Hospital	33
Hospital Geral Roberto Santos	21
Maternidade Anita Rodrigues Leal	20
Hospital do Oeste	20
Hospital Estadual da Criança	14

## DISCUSSION

Bahia was the third Brazilian state with the highest number of hospitalizations, registering 1,481 cases. The municipalities in Bahia with the highest prevalence were Itanhém, Salvador, and Barreiras, with 912, 445, and 20 cases, respectively.

Congenital hip deformities have an unknown etiopathogenesis, which can be attributed to mechanical factors such as delivery presentation. However, some risk factors, such as oligohydramnios, primiparous women, congenital knee recurves, and congenital muscular torticollis, may be screened during prenatal care, which indicate the correlation between physiological factors and/or their imbalances, namely, ligament laxity and hormonal changes (increased estrogen and relaxin) in women. In the social aspect, a relevant factor is the clothing of children with blankets that force the hip adduction position and the use of inappropriate diapers and straps can be risk factors for developing developmental dysplasia of the hip.^7^

In deliveries in which the fetus is arranged longitudinally within the uterus (breech presentation), the baby’s anatomy is distorted, with compression of the left hip against the mother’s sacral region, which generates a greater movement of adduction of the child’s hip to facilitate childbirth. In addition, extreme hip flexion with knee extension causes dislocation of the femoral head and leads to shortening and contracture of the iliopsoas muscle, which increases the likelihood of developmental dysplasia of the hip.^8^

Clinically, the diagnosis of developmental dysplasia of the hip in newborns and infants is performed using the Barlow and Ortolani maneuvers. A reduced but movable hip confers a positive Barlow maneuver. On the contrary, the Ortolani maneuver is positive when the hip is dislocated, but it is reducible.^9^ These maneuvers allowed the early detection of congenital hip deformities; however, some cases can only be detected with ultrasound since the instability of the coxofemoral joint, tested during the maneuvers, is not synonymous with dislocation.^9^

In infants older than 3 months, the clinical presentation of limited hip abduction and unilateral shortening of one of the lower limbs suggests developmental dysplasia of the hip. After gait exposure, some anatomical changes can be visualized during the physical examination, such as limited hip abduction, positive Trendelenburg, “anserine” gait, and lumbar hyperlordosis.^9^

Complementary imaging tests help in the diagnosis. Static and dynamic ultrasound is the test of choice. However, its limitations consist of being an operator-dependent test in addition to generating false-positive results.^10,11^

Simple radiography can also be used; however, due to bone development, newborns have a large part of cartilaginous tissue, which makes visualization and analysis difficult through this method, and is indicated only for babies over 4 months of age. Hip arthrography, computed tomography, and magnetic resonance are other possible tests to facilitate the diagnosis when well indicated.^12^

However, the data pointed out by the study prove that the diagnosis and the search for treatment have still occurred late in the state of Bahia. The city of Itanhém recorded 99.3% of cases of hospitalization for developmental dysplasia of the hip after 15 years of age; the other 0.7% were in the age group of 9–14 years. Meanwhile, in the capital of Bahia, 71.9% of the cases corresponded to the age group from 0 to 14 years, which configures a picture of better diagnostic assistance.^6^ In this context, it is possible to infer the difficulties encountered in several municipalities due to the lack of either qualified professionals for targeted assessment or resources to carry out complementary exams.

Even though Itanhém has a higher prevalence, the hospital costs recorded are lower than in other municipalities with lower numbers of cases. It is worth emphasizing the need to search for causes that directly justify lower spending in a region with higher incidence. It is speculated that there is a preponderance of regulations for reference centers due to the lack of resources in the city in question, less political investment, and the reduction of diagnoses due to a lack of specialists. Another crucial issue to be clarified is the predisposing factors found in this small municipality that put it in the first place among the congenital deformities of the hip joint in the state of Bahia.

Treatment of developmental dysplasia of the hip includes early diagnosis, joint reduction, and stabilization of the hip. The treatment division follows the diagnostic age group. In newborns, the femoral head is reduced in the acetabular cavity and its maintenance until joint stability is achieved with the use of the Pavlik brace, ensuring the reduction in flexion to avoid forced abduction.^13^

From 4 to 18 months of age, immediate abduction and subsequent reduction are performed under anesthesia with a tenotomy of the hip adductors. Soon after, a cast immobilization of the pelvicopodalic type is performed in the human Salter position for 6–8 weeks, and later, use of a Milgram-type orthosis for a period ≥2 months. Traction before the zenith has been a reason for the divergence in the literature, considering that it does not alter the treatment result. After 18 months of age, children are expected to start walking. At this age, the literature indicates immediate surgical intervention using the Salter osteotomy technique, ensuring anterolateral coverage of the femoral head.^8,13^

There are numerous surgical techniques for performing pelvic osteotomies to correct late acetabular dysplasia: preservation of articular cartilage, triple osteotomies (Steel technique), or medial sliding (Chiari technique). In which the frequency, degree of disability, duration of symptoms, morbidity, mortality, and osteonecrosis are considered, which is the most serious complication related to iatrogenesis in the treatment of developmental dysplasia of the hip.^13,14,15^ In Salter’s osteotomy, femoral shortening can be performed in parallel as an alternative to contain complications. However, some other complications can be installed. Like any invasive surgical procedure, it can be exposed to problems such as infections, secondary anatomical lesions, osteochondritis, dislocations, and avascular necrosis.^16,17^

Exposure of the hip joint with an abduction greater than 70º or forced medial rotation in treatment is a frequent cause of osteonecrosis. Therefore, conservative or surgical treatment (closed or open reduction technique) must comply with basic principles to reduce complications associated with poor performance. Another possible complication is vascular impairment of the femoral epiphysis, the bone area responsible for the joint, with varying degrees of severity, with or without injury to the proximal epiphyseal plate of the femur.^18,19^

Regarding functional rehabilitation, as in other orthopedic procedures, it is necessary to insert physical therapy treatment that allows an improvement in the patient’s quality of life. This is due to the evolution of science, with the application of biomechanical techniques aimed at gait training that should be started as soon as the child can stand and can be associated with the hydrotherapy technique, resulting in better neuromuscular recovery.^3^

The analyzed data collection platform (DATASUS) is an instrument that has limitations since the data presented do not allow us to reliably observe the relationship between cause and effect. Thus, it is not possible to guarantee the effectiveness of public policies, although they allow for the consistent analysis of numerous aspects involving public health in Brazil, making them an effective instrument for scientific validation.

From the analysis of the data obtained, it was possible to observe the relationship between congenital deformities of the hip and the epidemiological situation in the state of Bahia. The high prevalence of mixed-race women and the predominance in some cities, such as Itanhém and Salvador, are the factors to be highlighted, as it is a problem with a low mortality rate but a high potential for compromising the individual’s quality of life. In this context, it appears that this public health issue still needs investments for better diagnostic assistance in some municipalities, since the late diagnosis of congenital hip deformities is still a reality even with several clinical and imaging methods available, which makes treatment and rehabilitation of the patient difficult when compared to an early diagnosed condition.

## Data Availability

The database that originated the article is available with the corresponding author.
